# Personalized contact strategies and predictors of time to survey completion: analysis of two sequential randomized trials

**DOI:** 10.1186/1471-2288-15-5

**Published:** 2015-01-09

**Authors:** Victor D Dinglas, Minxuan Huang, Kristin A Sepulveda, Mariela Pinedo, Ramona O Hopkins, Elizabeth Colantuoni, Dale M Needham, the NIH NHLBI ARDS Network

**Affiliations:** Outcomes After Critical Illness and Surgery (OACIS) Group, Johns Hopkins University, 1830 E Monument Street, 5th floor, 21205 Baltimore, MD USA; Division of Pulmonary and Critical Care Medicine, Department of Medicine, Johns Hopkins University, 1830 E Monument Street, 5th floor, 21205 Baltimore, MD USA; Pulmonary and Critical Care Division, Department of Medicine, Intermountain Medical Center, 5121 Cottonwood Street, 84157 Murray, UT USA; Psychology Department and Neuroscience Center, Brigham Young University, 84602 Provo, UT USA; Department of Biostatistics, Bloomberg School of Public Health, Johns Hopkins University, 615 N Wolfe Street, 21205 Baltimore, MD USA; Department of Physical Medicine and Rehabilitation, Johns Hopkins University, 1830 E Monument Street, 5th floor, 21205 Baltimore, MD USA

**Keywords:** Randomized controlled trial, Survey methods, Respiratory distress syndrome, Adult, Mail, Telephone, Cohort studies

## Abstract

**Background:**

Effective strategies for contacting and recruiting study participants are critical in conducting clinical research. In this study, we conducted two sequential randomized controlled trials of mail- and telephone-based strategies for contacting and recruiting participants, and evaluated participant-related variables’ association with time to survey completion and survey completion rates. Subjects eligible for this study were survivors of acute lung injury who had been previously enrolled in a 12-month observational follow-up study evaluating their physical, cognitive and mental health outcomes, with their last study visit completed at a median of 34 months previously.

**Methods:**

Eligible subjects were contacted to complete a new research survey as part of two randomized trials, initially using a randomized mail-based contact strategy, followed by a randomized telephone-based contact strategy for non-responders to the mail strategy. Both strategies focused on using either a personalized versus a generic approach. In addition, 18 potentially relevant subject-related variables (e.g., demographics, last known physical and mental health status) were evaluated for association with time to survey completion.

**Results:**

Of 308 eligible subjects, 67% completed the survey with a median (IQR) of 3 (2, 5) contact attempts required. There was no significant difference in the time to survey completion for either randomized trial of mail- or phone-based contact strategy. Among all subject-related variables, age ≤40 years and minority race were independently associated with a longer time to survey completion.

**Conclusion:**

We found that age ≤40 years and minority race were associated with a longer time to survey completion, but personalized versus generic approaches to mail- and telephone-based contact strategies had no significant effect. Repeating both mail and telephone contact attempts was important for increasing survey completion rate.

**Trial registration:**

NCT00719446.

## Background

Underpowered studies and insufficient sample sizes often result, in part, from ineffective participant contact methods and associated poor recruitment and participation rates [1, 2]. Unsuccessful participant contact leads to extended recruitment time, missing data, and increased cost and other resource utilization [3]. Nearly 60% of randomized controlled trials had difficulties in meeting recruitment target or needed to extend recruitment period [4]. Achieving a timely and efficient strategy to contact research participants is critical for conducting clinical research [4].

Several studies have shown that participants who were female, Caucasian, younger, more educated, and employed tend to have faster response to surveys [5–9]. However these findings are not universally confirmed, with conflicting results regarding factors, such as participant demographics and health status, influencing participant recruitment and contact [10–18]. Some of these conflicting results may be due to different patient populations studied and different countries of research. Hence, we aimed to evaluate predictors of survey completion in a patient population and U.S. study setting similar to prior research by Chen et al. [13]. Chen et al. [13] studied 146 acute lung injury (ALI) survivors from a single-center, multi-site longitudinal observational study based in the U.S.

Building on the results of Chen et al. [13], in the current study, we evaluated a similar population of ALI survivors, but with a larger sample size of 332 participants, recruited from 41 hospital sites at 12 centers across the U.S. Like Chen et al., this trial was conducted with participants who had previously been enrolled in an observational follow-up study evaluating physical and mental health outcomes. In our current study, the last research evaluation occurred a median of 34 months previously. As part of this study, we asked participants to complete a new one-page insurance survey that was not part of the evaluation in their prior research visits.

The current study was conducted via two sequential randomized controlled trials evaluating the effects of different mail- and then telephone-based strategies for contacting and recruiting participants, and evaluated participant-related variables associated with time to completing the survey. As commonly done in survey-based research, the study protocol was designed with initial participant contact via mail, with more resource-intensive telephone contact reserved for non-responders to the mailed survey [13, 19–21]. Based on the results of prior research [22–24] and the non-statistically significant signal favoring personalized form of communications observed in the smaller-sized study by Chen et al. [13], we hypothesized that using personalized contact strategies, such as hand-written envelopes for mail and using a specific investigator’s name for telephone messages, would result in a faster time to completing the survey and a higher response rate. We also hypothesized that for participants with poorer physical and mental health status, at the time of last research contact, the time to survey completion would be longer.

## Methods

### Study population

Participants in these two sequential randomized trials of mail- and telephone-based contact strategies were part of the ARDSNetwork Long Term Outcomes Study (ALTOS) [25, 26], which evaluated 6 and 12 months patient outcomes in acute lung injury survivors who were enrolled in randomized trials of novel interventional therapies funded by the National Heart, lung, and Blood Institute (NHLBI) ARDS Network [27, 28]. A new, brief, one-page health insurance survey was introduced part-way through ALTOS. A total of 332 ALTOS participants, who had been enrolled prior to introduction of this survey, were sequentially randomized to mail- and then telephone-based (for mail non-responders) strategies, if needed, for contacting participants to complete the survey. This study was approved by the Institutional Review Board at Johns Hopkins University. Written or oral informed consent was obtained from all participants in the study.

### Standardized protocol for contacting participants

Within our two sequential randomized mail and telephone trials of contact strategies, conducted from June to December 2012, eligible subjects were contacted using a multi-step, structured protocol starting with mailed letters and then telephone calls, as needed for non-responders to mailings. In the mail trial, participants were mailed the insurance survey every 2 weeks until the survey was completed or the participant was sent a total of 4 mailings. For these mailings, trial participants were randomized to receive either a “personal format letter” in which their mailing address and the return address were hand written and a traditional stamp was stamped using the envelope versus a “business format letter” in which the addresses were typed and the postage was affixed by a commercial stamp-machine. In all other respects, the envelopes were identical (i.e. 9 × 12 inch manila envelopes) and included an identical cover letter, insurance survey, and self-addressed return envelope.

Starting 20 days after the end of the mail trial, a telephone trial was initiated. Non-responders from the prior mail trial and those excluded from mail trial due to lack of a correct mailing address were eligible for the telephone trial. The telephone trial focused on the type of messages left for participants via either an answering machine/voicemail or a person (other than the participant) answering the telephone. In this trial, participants were randomized to receive either a generic message in which the caller said that she was “calling on behalf of the ARDS Network Long-Term Outcomes study”, or a personalized “ALTOS principal investigator message” where the caller said she was “calling on behalf of Dr. Dale Needham.” These telephone calls were made once weekly by the same caller, for up to 4 weeks, until the participant was reached by telephone or the participant called back and completed the survey. If the participant answered the telephone and completed the survey with no message ever left, the participant was excluded from analysis of the telephone trial since they were not exposed to the telephone message intervention. In both the mail and the phone trials, randomization was performed by a statistician using computer-generated random numbers with an allocation ratio of 1:1.

### Measurement of outcome and predictor variables

The primary outcome for analysis was the time (in days) for the participant to complete the insurance survey, analyzed separately for the sequential mail and telephone trials. Given the nature of this study design, outcome assessment was not blinded, but participants were blinded. In the mail trial, time zero was defined as the first mailing, using a valid address, to the participant. For the telephone trial, time zero was the date of the first telephone message to the participant. Given a lack of significant differences between the randomized groups in both the mail and telephone trial (see Results section), the participants were subsequently pooled into a single cohort to evaluate the association between the time to survey completion and potential predictors, using time zero from the mail trial for all participants (except for those without a valid mailing address in which time zero was defined as the start of the phone trial). A total of 18 potential predictors of time to survey completion were evaluated in this pooled analysis, grouped into two categories: (1) participant baseline demographics and time (in months) since last follow-up with the ALTOS study, and (2) physical and mental health status at last follow-up. Participants’ baseline demographic factors evaluated were: age, sex, race, and employment status. Participants’ physical and mental health status factors evaluated were: physical functioning (using the Functional Performance Inventory-Short Form (FPI) [29, 30] total score and the subscale scores for body care, physical exercise, and maintaining household [for each, range 0–3; higher score is better]), quality of life (using the Short Form-36 version 2 (SF-36) [31] physical and mental component scores [range 0–100; higher score is better], as well as EuroQol five dimensions questionnaire (EQ-5D) [32, 33] visual analogue scale score (VAS) [range 0–100; higher score is better] and utility score [range -0.11-1.0; higher score is better]), cognition (using Mini-Mental State Examination (MMSE) telephone version converted score [34] [range 0–30; higher score is better]), mental health (using the Hospital Anxiety and Depression Scale (HADS) [35] subscale scores for anxiety and for depression [for each, range 0–21; lower score is better], as well as Impact of Event Scale-Revised score (IES-R) [36, 37] for post-traumatic stress disorder symptoms [range 0–4; lower score is better]), and fatigue (using the Functional Assessment of Chronic Illness Therapy (FACIT) [38, 39] fatigue interval scale score [range 0–100; higher score is better]).

### Statistical analysis

We compared participant demographics and physical and mental health status among participant groups in each of the sequential mail and the telephone randomized trials using Fisher’s exact test (categorical variables) and t-tests (continuous variables). P-values for difference in survey completion rates between the two randomized groups in each of the mail and the telephone trials were calculated using two-sample test of proportions. The distribution of time to survey completion was compared using Kaplan-Meier survival estimates and log-rank tests. The associations between participant demographics, time since last contact with participant, and physical and mental health status with time to survey completion were evaluated using bivariable hazard ratios (HRs) from simple Cox proportional hazards regression models, and then evaluated via adjusted hazard ratios in multivariable Cox models. Since we had 183 responders and 18 predictors, the ratio of the total number of responding participants to the number of potential predictors was greater than 10 [40, 41], so all variables were included in our multivariable model without substantial concern for overfitting. Moreover, to further demonstrate no overfitting of the model, we sequentially removed each of the two major categories of predictor variables from the model and confirmed no material change in the remaining variables’ associations with time to survey completion.

We confirmed that there was no violation of the proportional hazards assumption by examining Schoenfeld residual plots for each exposure and confirmed that there was no multi-collinearity by evaluating variance inflation factors [42, 43]. Outliers and influential points were assessed using Dfbeta statistics [44]. We verified the linearity assumption for each continuous predictor variable, by assessing plots of the variable versus Martingale residuals from the Cox model. Only participant age demonstrated a non-linear relationship with time to survey completion, resulting in dichotomizing age as ≤40 versus >40 years old. All p-values were two-sided, and statistical significance was defined as p <0.05. All statistical analyses were performed using Stata 13.0 (StataCorp, College Station, TX).

## Results

### Overall recruitment

Participants in the study had a mean age of 49 years old, 52% women, 80% white, with 70% retired, disabled or unemployed at their last study contact, with similar characteristics between randomized groups (Table 1).

**Table 1:** **Baseline characteristics of study population by treatment groups in mail and telephone randomized trials**

	Mail trial			Telephone trial		
Characteristics ^1^	All participants (N = 332)	Personalized envelope (N = 166)	Business envelope (N = 166)	All participants (N = 171)	Study name in message (N = 86)	P.I. name in message (N = 85)
**Participant demographics and last contact**						
Age, mean (SD)	48.8 (15.0)	48.2 (15.4)	49.4 (14.7)	46.0 (14.7)	46.7 (14.0)	45.3 (15.5)
Male, n (%)	160 (48)	77 (46)	83 (50)	84 (49)	44 (51)	40 (47)
Minority^2^, n (%)	64 (20)	27 (17)	37 (23)	42 (26)	19 (23)	23 (28)
Employment status^3^						
Employed (full- or part-time), n (%)	97 (30)	41 (25)	56 (34)	52 (31)	32 (38)	20 (24)
Unemployed, n (%)	79 (24)	43 (26)	36 (22)	43 (26)	15 (18)	28 (34)
Retired or disabled, n (%)	151 (46)	80 (49)	71 (44)	72 (43)	37 (44)	35 (42)
Months since last contact, mean (SD)	33.7 (7.3)	34.2 (7.2)	33.3 (7.4)	33.5 (7.5)	35.2 (7.1)	31.7 (7.5)
**Physical and mental health status** ^**3**^ **, mean (SD)**						
FPI overall score	2.0 (0.7)	2.0 (0.7)	2.0 (0.7)	1.9 (0.7)	1.9 (0.8)	2.0 (0.7)
FPI body care subscale score	2.5 (0.6)	2.5 (0.6)	2.5 (0.6)	2.5 (0.7)	2.4 (0.7)	2.6 (0.6)
FPI maintaining household subscale score	1.9 (0.9)	1.9 (0.9)	1.9 (0.9)	1.9 (0.9)	1.8 (1.0)	1.9 (0.8)
FPI physical exercise subscale score	1.6 (0.9)	1.6 (0.9)	1.7 (0.9)	1.6 (0.9)	1.6 (1.0)	1.6 (0.9)
SF-36 Physical Component Summary	40.5 (12.5)	40.7 (12.4)	40.3 (12.7)	40.5 (12.8)	40.0 (13.3)	41.1 (12.3)
SF-36 Mental Component Summary	44.9 (14.8)	45.0 (14.4)	44.6 (15.2)	42.1 (14.8)	41.4 (14.3)	42.9 (15.3)
EQ-5D visual analogue scale	69.1 (22.7)	70.3 (22.3)	67.9 (23.0)	67.4 (23.7)	65.5 (24.2)	69.2 (23.3)
EQ-5D utility score	0.7 (0.2)	0.7 (0.2)	0.7 (0.2)	0.7 (0.3)	0.7 (0.3)	0.7 (0.2)
MMSE score	25.6 (1.9)	25.5 (2.0)	25.7 (1.8)	25.5 (2.2)	25.4 (2.6)	25.6 (1.7)
HADS-depression subscale score	6.0 (4.9)	5.9 (4.9)	6.2 (5.0)	6.7 (4.9)	6.8 (5.1)	6.6 (4.7)
HADS-anxiety subscale score	7.0 (5.1)	7.0 (5.3)	7.1 (4.9)	8.0 (5.0)	7.9 (4.7)	8.0 (5.2)
IES-R score	1.0 (1.0)	1.0 (1.0)	1.0 (1.0)	1.1 (1.0)	1.1 (1.0)	1.2 (1.0)
FACIT score	62.3 (18.6)	62.3 (20.0)	62.2 (17.1)	60.2 (16.9)	59.4 (15.6)	61.1 (18.0)

*Abbreviations:* *SD* (Standard Deviation), *FPI* (Functional Performance Inventory), *SF-36* (Short Form-36 Health Survey), *EQ-5D* (EuroQol Five Dimensions Questionnaire), *MMSE* (Mini-Mental State Examination), *HADS* (Hospital Anxiety and Depression Scale), *IES-R* (Impact of Event Scale-Revised), *FACIT* (Functional Assessment of Chronic Illness Therapy).

^1^Missing values for each variable: months since last contact (1, 1), white (12, 7), employment (5, 4), all FPI scores (10, 7), SF-36 Physical and Mental Component Summaries (17, 10), EQ-5D VAS (10, 7), EQ-5D utility (9, 6), MMSE (20, 12), HADS subscales (14, 9), IES-R (16, 11), FACIT (16, 10).

^2^Minority race includes African American, Asian, American Indian and Alaskan Native.

^3^Status as at last contact with research participant.

### Randomized interventions

**Figure 1:**
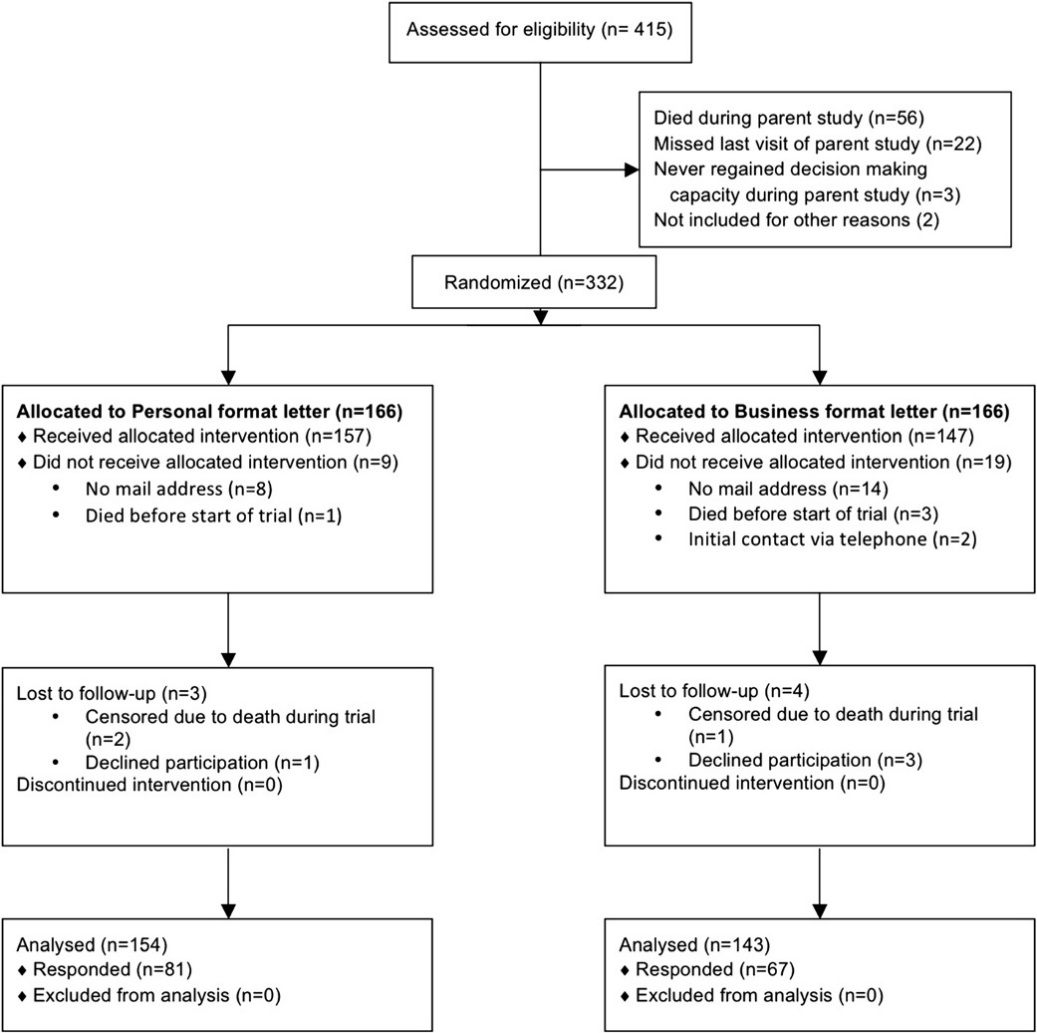
**CONSORT flow diagram for mail trial.** Mail survey completion rate: (81 + 67) respondents/(157 + 147) potential respondents = 49%.

**Figure 2:**
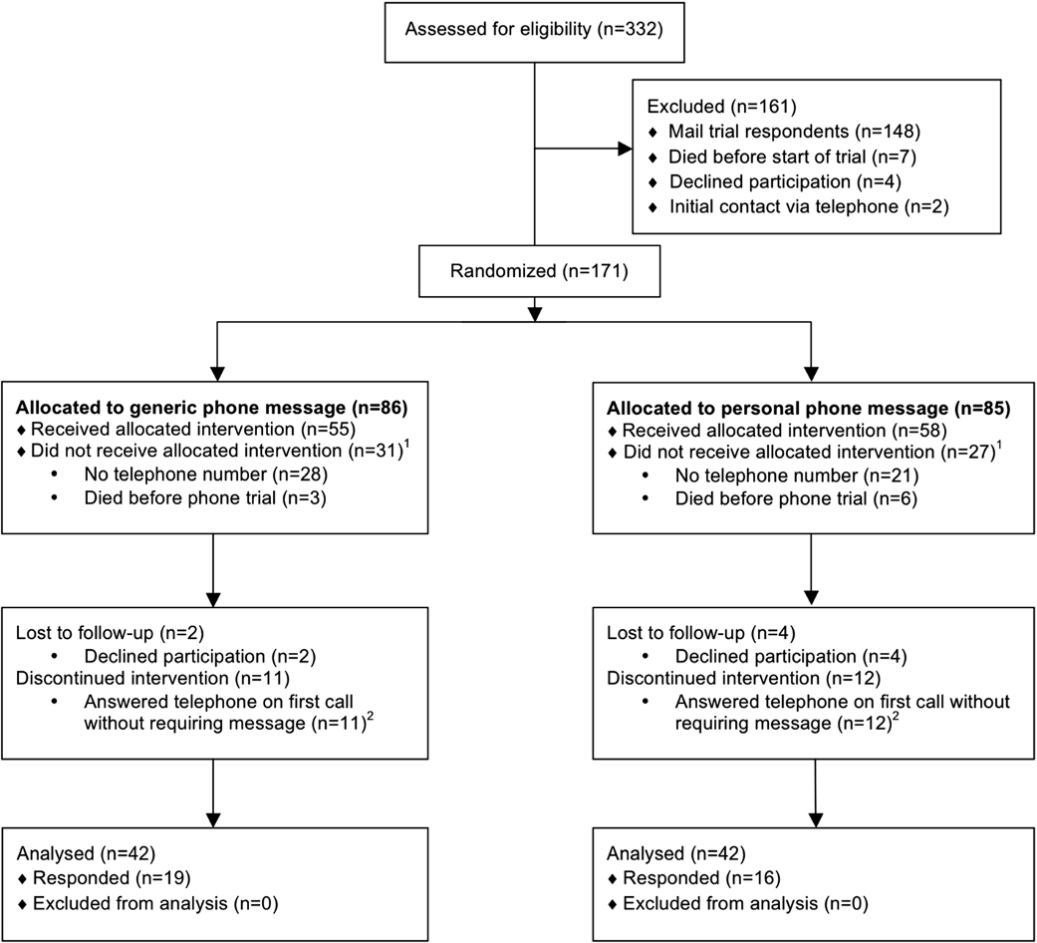
**CONSORT flow diagram for phone trial.** Telephone survey completion rate: [(19 + 16) respondents + (11 + 12) answered telephone on first call]/(55 + 58) potential respondents = 51%. ^1^ Excluded from analysis of randomized trial of telephone messages, but censored in analysis of the entire cohort. ^2^ Included in calculation of survey completion rate, but excluded from Cox models.

There are 332 subjects potentially eligible for the mail trial (Figure 1) who were sent either personal (n = 166, 50%) or business (n = 166, 50%) format letters. After excluding a total of 32 subjects not eligible to respond and therefore not receiving the allocated intervention (Figure 1), a total of 148 (49%) of 300 eligible subjects completed the survey with the mail trial, of which 81 (52%) were in personal format group and 67 (47%) were in business format group (p = 0.35). For the telephone portion of the trial (Figure 2), 171 eligible subjects who did not respond to the mail trial or had no valid mailing address were contacted by telephone, of which 86 (50%) were in the generic “ALTOS study” telephone message group and 85 (50%) in personalized “ALTOS principal investigator” message group. Among 84 participants exposed to randomized phone message and eligible to respond in the telephone trial, 35 (42%) completed the survey, of which 19 (45%) were in the ALTOS study telephone message group and 16 (38%) were in ALTOS principal investigator message group (p = 0.51).

**Figure 3:**
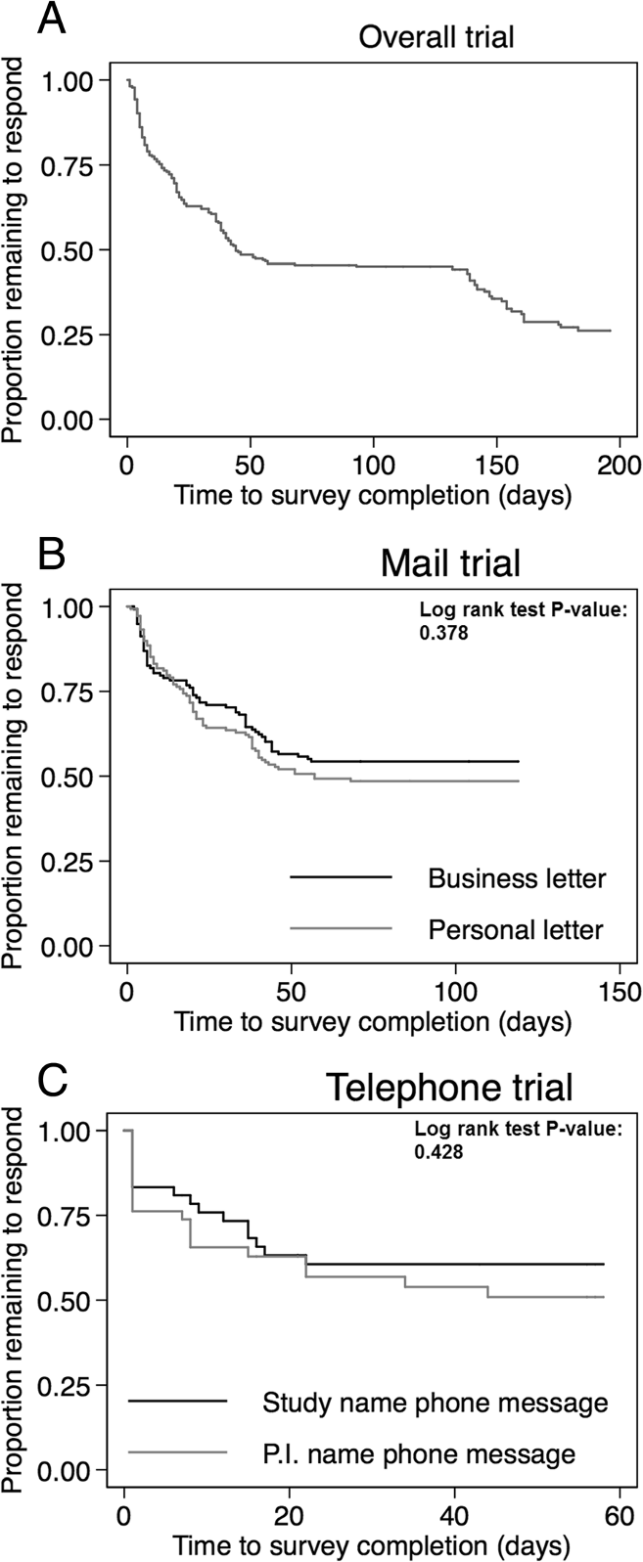
**Estimated proportions of participants remaining to be contacted over time.** These 3 panels display the survival function for time to survey completion since randomization for the mail- and telephone-based randomized trials. Panel **A** displays the overall survival function for all participants participating in the trials. Panels **B** and **C** display the survival function for the mail and telephone randomized trials, respectively.

Figure 3 shows the Kaplan-Meier curves for time to survey completion for the entire cohort (Panel A), and for the mail trial (Panel B) and the telephone trial (Panel C). There was no significant difference in median (inter-quartile range, IQR) time to survey completion between personal versus business format letter at 18 (7–38) versus 18 (5–36) days, with a bivariable hazard ratio (HR) (95% confidence interval [CI]) of 1.16 (0.83-1.62, p = 0.383). There was also no significant difference in median (IQR) time to survey completion between generic “ALTOS study” versus personalized “ALTOS principal investigator” message group at 7 (1–15) versus 7 (1–15) days, with a bivariable HR (95% CI) of 1.29 (0.66-2.51, p = 0.455). In multivariable analyses these associations remain non-significant for both the mail (HR, 95% CI: 1.05, 0.73-1.50, p = 0.797) and telephone (HR, 95% CI: 1.23, 0.50-2.99, p = 0.644) trials. Among those who completed surveys, the median (IQR) number of contact attempts was 2 (1, 3) in mail trial and 1 (1, 3) in telephone trial.

### Pooling of participants from randomized groups

Overall, when pooling participants from the two trials (n = 332), after excluding 16 (5%) participants who died and 8 (3%) who had no mailing address and active telephone number for contacting the participant, there were 308 subjects eligible to respond, of whom 206 (67%) responded and 10 (3%) declined to complete the survey. Responders required a median (IQR) number of 3 (2, 5) contact attempts (including both mail and/or telephone) before survey completion.

### Predictors of time to survey completion in pooled participant cohort

Within the analysis of the pooled group of participants, in simple Cox regression models (Table 2), 5 predictors were significantly associated with time to survey completion: younger age, minority race other than white (including African American, Asian, American Indian and Alaskan Native), and three measures of mental health (SF-36 MCS, HADS-Anxiety subscale, and IES-R PTSD symptom score). In multivariable Cox regression analysis, only 2 variables were independently associated with a significantly longer time to survey completion: (1) age ≤40 years old (HR, 95% CI: 0.62, 0.41-0.95) and (2) minority race (HR, 95% CI: 0.58, 0.36-0.93).

**Table 2:** **Bivariable and multivariable associations of baseline characteristics with time to survey completion in entire cohort**

	Bivariable model		Multivariable model	
Predictors	HR (95% CI) ^1^	P-value	HR (95% CI) ^1^	P-value
**Participants demographics and last contact**				
Age ≤40 years old	**0.64 (0.45, 0.92)**	**0.016**	**0.62 (0.41, 0.95)**	**0.029**
Male	0.86 (0.64, 1.16)	0.315	0.70 (0.49, 1.01)	0.052
Minority^2^	**0.55 (0.35, 0.84)**	**0.006**	**0.58 (0.36, 0.93)**	**0.023**
Employment status^3^				
Employed (full- or part- time)	Ref		Ref	
Unemployed	0.95 (0.62, 1.44)	0.789	1.11 (0.71, 1.74)	0.653
Retired or disabled	1.27 (0.89, 1.79)	0.184	1.31 (0.83, 2.09)	0.248
Month since last contact	1.00 (0.98, 1.02)	0.869	0.99 (0.97, 1.01)	0.465
**Physical and mental health status** ^**3**^				
FPI overall score	1.10 (0.88, 1.37)	0.418	0.84 (0.45, 1.56)	0.584
FPI body care subscale score	1.26 (0.96, 1.65)	0.099	1.27 (0.77, 2.10)	0.350
FPI maintaining household subscale score	1.06 (0.89, 1.26)	0.532	1.08 (0.71, 1.65)	0.709
FPI physical exercise subscale score	1.07 (0.90, 1.26)	0.443	1.13 (0.79, 1.62)	0.497
SF-36 Physical Component Summary	1.00 (0.99, 1.01)	0.805	0.99 (0.96, 1.02)	0.487
SF-36 Mental Component Summary	**1.01 (1.00, 1.03)**	**0.010**	1.01 (0.99, 1.04)	0.262
EQ-5D visual analogue scale	1.00 (0.99, 1.01)	0.201	1.00 (0.99, 1.01)	0.607
EQ-5D utility score	1.41 (0.75, 2.66)	0.290	0.97 (0.25, 3.70)	0.960
MMSE score	1.01 (0.92, 1.11)	0.835	0.95 (0.85, 1.07)	0.420
HADS-depression subscale score	0.97 (0.94, 1.00)	0.067	1.02 (0.95, 1.09)	0.445
HADS-anxiety subscale score	**0.96 (0.93, 0.99)**	**0.008**	0.99 (0.93, 1.06)	0.810
IES-R score	**0.84 (0.71, 0.99)**	**0.040**	1.07 (0.79, 1.45)	0.652
FACIT score	1.01 (0.99, 1.02)	0.068	1.01 (0.99, 1.03s)	0.223

*Abbreviations:* *SD* (Standard Deviation), *HR* (Hazard Ratio), *CI* (Confidence Interval), *FPI* (Functional Performance Inventory), *SF-36* (Short Form-36 Health Survey), *EQ-5D* (EuroQol Five Dimensions Questionnaire), *MMSE* (Mini-Mental State Examination), *HADS* (Hospital Anxiety and Depression Scale), *IES-R* (Impact of Event Scale-Revised), *FACIT* (Functional Assessment of Chronic Illness Therapy).

^1^A hazard ratio (HR) < 1 indicates a longer time to contact with the participant. All the significant associations (p<0.05) in models are highlighted in bold.

^2^Minority race includes African American, Asian, American Indian and Alaskan Native.

^3^Status as at last contact with research participant.

## Discussion

We conducted two sequential randomized controlled trials to evaluate separately the effects of personalized versus generic mail- and telephone-based contact strategies and also evaluated other potential predictors of time to participant survey completion for 332 ALI survivors recruited from 41 hospitals across the U.S. There was no significant difference in time to survey completion or response rate between a “personal” versus generic “business” style of envelope in the mail trial, or for a generic telephone message (that used the study name) versus a personalized telephone message (that used the principal investigator’s name) for non-responders to the mail trial. Among 18 demographic and physical and mental health status variables evaluated, only younger age (≤40 years old) and minority (vs. white) race were independently associated with a significantly longer time to survey completion within this sequential protocol of repeated mail then telephone contact strategies.

A multi-modal contact strategy, such as mail followed by telephone calls, and making repeated contact attempts, as done in this study, is typical of rigorous approaches for minimizing participant loss to follow-up and associated missing data [19–21]. In our study, this approach involved active contact for up to 12 weeks (maximum of 4 bi-weekly mailings and 4 weekly telephone calls) with participants who were last contacted approximately 3 years earlier. Via this approach, we achieved an overall survey completion rate of 67%, with only 3% declining participation, and required a median (IQR) of 3 (2, 5) contact attempts among respondents. This combined 67% survey completion rate, with individual response rates of 49% in the mail trial and 51% in the telephone trial, compares favorably with other studies [10, 22, 45]. Comparison of response rates between the mail versus telephone trials within our study was not conducted since participants in telephone trial represented non-responders from mail trial or participants without a valid mailing address, and thus a different population from the mailing trial. Contrary to our hypotheses developed based on prior studies [13, 22–24], we did not observe a significantly shorter time to survey completion or a higher response rate with more personalized contact formats, such as a personal envelope format and a telephone message using a the principal investigator’s name (“Dr. Dale Needham”).

Relatively few studies have evaluated factors predicting time to participant survey completion [10, 46]. Tennant and Badley [46] evaluated age as a single predictor for non-response bias, while Chen et al. [13] did not explicitly evaluate the effects of participants’ objective physical and mental health status. Although Chen’s study [13] found no association of any participant demographics with time to survey completion, we found significant associations for age and race. Our study demonstrated that participants ≤40 years old had a longer time to survey completion. This finding is similar to the results of Tennant and Badley [46] who demonstrated that participants ≤65 years old who were physically independent had a tendency to slower survey completion. We speculate that younger patients, especially after acute lung injury, may be less physically impaired and spent less time at home, making them less readily available to respond [25, 26, 47, 48]. Alternatively, expanded use of mobile phones among younger adults may make them more responsive to telephone versus mail communication; thus, the slower time to survey completion for younger patients may be a result of initial contact attempts being made via mail. Another potential explanation is that younger participants may have changed addresses more frequently than older participants. In addition, our study demonstrated that although several predictors of physical and mental health status had a significant association with time to survey completion in bivariable Cox models, they were not significant in multivariable analyses. Since the last study contact, for purposes of evaluating health status, was approximately 3 years earlier, this timing issue may have contributed to a lack of effect; however, specifically in ALI survivors, physical and mental health impairments are long-lasting [47, 49–51] which justified their evaluation in this study.

The median (IQR) number of contact attempts was 2 (1, 3) in the mail trial among those participants who completed the survey, which demonstrates that repeated attempts within a single contact modality are needed for survey completion. However, we observed a decreasing rate of survey completion over time in the mail and telephone trials (Figure 3), indicating that with repeated failed contact attempts, non-responders were less likely to respond. However, after changing from mail to telephone contact, additional participants were successfully contacted, with 51% of eligible participants completing the survey with a median (IQR) of 1 (1, 3) telephone calls among respondents. This finding suggests that the effects of single contact method (e.g. mail), despite some improvement with repetition, may be not adequate for timely and maximal participation. Utilizing at least two different types of contact strategies can increase participant response rate in clinical studies.

There are several potential limitations in our study. First, our study focuses on survivors of ALI, so our findings may or may not be generalizable to other specific populations. However, this research was a national study of participants initially recruited from 41 hospitals in United States, so our findings may be generalizable to similar populations across the U.S. Second, there was a lag between the participant’s last visit at the end of the ALTOS study and our attempt to contact them in this trial, so their employment and physical and mental health status may have changed over time, impairing our ability to identify the true association between these variables and survey completion. Third, our analyses may have omitted potentially relevant predictor variables that were not available for analysis in this study. For instance, Chen et al. [13] evaluated factors related to prior research visits (e.g., incomplete data collection and missed visits), and revealed significant associations with participant contact. Moreover, other comorbidities and disease history, not evaluated in this study, may be related to timing of survey completion. Fourth, the difference between our contact strategies (generic versus personal) may have been too small to yield a significant difference in participant perceptions and response times. However, we felt that our interventions were not markedly different from prior studies [22–24] that demonstrated a substantial difference in response rates between personal and generic contact strategies. Additionally, it’s possible that the personalized contact strategy would, in fact, shorten response time had these participants not already been enrolled in a lengthy study. However, their prior involvement may not have played a large role since this trial was conducted 3 years after their last follow-up visit. Lastly, the questionnaire mailed was a single-page, retrospective survey of insurance coverage status. Perhaps faster response time and higher responses rates would have been achieved, in the mailing portion of the trial, if the survey was more intriguing to the participant.

## Conclusion

Repeated attempts using both mail- and telephone-based contact strategies are important for effectively reaching a majority of ALI survivors for completion of a simple, one-page health insurance survey. More personalized contact strategies (e.g. personalized format letter, and use of an investigator’s name in telephone messages) were not associated with a significantly faster time to survey completion. Although participants’ prior employment and physical and mental health status were not independently associated with a shorter time to survey completion, participants who were minority and aged under 40 years old had a significantly longer time to survey completion. Greater contact efforts and novel investigation of contact methods are needed for maximizing survey completion rates, especially for younger participants and racial minorities.
